# Dr. Kinglake's Reply to Constant Reader, on Gout

**Published:** 1804-03-01

**Authors:** Robert Kinglake

**Affiliations:** Taunton


### *To the Editors of the Medical and Physical Journal.*

GENTLEMEN,

IT is my endeavour to observe as a regulating maxim in my conduct, *Fac nihil per iracundiam*; nor shall my anger even be inordinately excited by the renewed and unprovoked attack of "*Constant Reader*,"* envenomed as it is with Horatian satire, "*bilious*" acrimony, and disappointed wit.

Such weapons are, in my estimation, never respectable, and when wielded in the clumsy and misdirected manner of your "shrouded" correspondent, are more than pointless.

My refutation of "*Constant Reader's*" mis-statement†, was that which truth required; and the mode in which it was done, had too much of urbanity, either to "*move bile* or justify ridicule.

"Tolle jocos non est jocus esse malignum."

My want of triumph, however, is "*Constant Reader's*" exultation. But why this revengeful satisfaction? Does he regret that the *truth* is discovered? Was he actuated by either a *suggestio falsi* or *suppressio veri*, with respect to the unfounded report. This is indeed a malicious subterfuge, and much too suspicious, to be consistent with the sincere regard he professes for medical improvement, and humanity.

It is admitted that death took place in the case alluded to, but does sudden death never happen but from gouty diathesis? Are not the violent and manifold influence of vascular plenitude, obstruction, rupture, and convulsive actions, occurring in the vital organs, sufficient to account for sudden death, without gratuitously referring to a ghostly something, called *gouty diathesis*? The only diathesis allowable to "*Constant Reader*," on this occasion, is that of revenge, for having been detected in attempting to authenticate a false rumour.

In very few words my vindication shall be restated, from all the reproachful, illiberal, and unmanly insinuations of "*Constant Reader*," relative to my claiming an originality in

* See Med. and Phys. Journal, No. 60, p. 150—1. † Ibid. No. 58, p. 500—2.

in the use of cold water in gout, and then let the public impartially say, what is *offensive cavilling*, and what is an *original* and *honest* endeavour to improve practical medicine. The ground on which cold water, or reduced temperature, was proposed by me as a safe and effectual remedy for gout, was founded on a radical resemblance, which appeared to me to subsist between gouty and every other description of inflammation, that they were also in commencement of local, and not of constitutional origin, and that, by parity of reason, they ought to yield to similar treatment.

That this was my view of the subject, is verified by the following quotation from my former reply to "*Constant Reader*," published in the Medical and Physical Journal.*

"It has never been my object to assume credit for either originality or peculiarity, in reducing the morbid excess of temperature in arthritic affection, by diminished heat. It is impossible the principle should have escaped the earliest reasoning on the subject. The principle of the practice may therefore be rather considered as *common* to human intelligence than *peculiar* to any individual. The doctrine of distempered heat at once pervades and constitutes the most intelligent and instructive parts of Hippocrates's writings. The medical principles and practice of Sydenham also, founded on temperature, formed a transcendant epocha in the history of curative medicine, and happily for mankind, finally overthrew the fatal delusions of humoral pathology, and alexipharmic jargon. Conducted then by analogy, it occurred to me as highly reasonable, that gout, distinguished like other inflammations by excessive heat, and marked by no essential difference, might be subdued in a similar manner. This persuasion induced me to assimilate the treatment. Not a single fact had previously reached my knowledge to authorise the trial; though, undoubtedly, many were extant. To me therefore the practice was relatively, though not absolutely, original. The only claim to originality which seems to exist in my right, and the only one which deserves a moment's solicitude to establish, is that of publicly recommending the practice, after having experienced its salutary effects in numerous instances, in which the treatment was conducted with such disguise and secresy, as were necessary to obviate the prohibitive influence of prevailing prejudices against it."

The

* See Med. and Phys. Journal, No. 51, p. 442—4.

The shelves of neither ancient nor modern authors offered any thing that suited the object of my inquiry. It would be in vain to search the stores of medical literature for any authority to countenance my principle, that gout is exclusively an inflammatory affection of the ligamentous and tendinous structure, that it is merely local,* and unknown as a constitutional complaint, but through the medium of morbid sympathies, generated and rendered more or less inveterate by its protracted duration. The originality professed by me was, that of decidedly recommending the practice to public adoption; not because it merely afforded alleviation to pain, but because such alleviation of pain was justified by the nature of the disease in being a local and not a constitutional affection. My claim to the refrigerant treatment of gouty inflammation is not rested on so baseless a pretence, as that of having been the first casually to have subjected to trial the topical use of cold water, but on the sure foundation of having justified, applied, and realised its indisputable safety and efficacy by a theory † which undauntedly aspires to be at once perfectly original and rational.

The opinion which has been hitherto held of gout, must have ever opposed an insuperable obstacle to a scientific adoption of reduced temperature for its cure, however glaring its incidental efficacy might have appeared, or however strongly recommended. The establishment of the remedy therefore was impossible, without the originality which is in my full (it may be presumed indisputable) right, that of considering gout as a *local* affection, wholly independent of *constitutional* origin.

Neither my medical reading nor conversation had furnished the smallest clue to forming my opinion, which led me so far to neglect what had generally been written on gout, as to have overlooked all and every of those *splendid authorities* quoted by "*Constant Reader*" for the topical use of cold water in that disease. These quotations are totally useless and quite irrelevant to the purpose for which they are adduced. They say no more than may be attested by the experience of many hundred individuals, that gouty pain may be subdued by the external application of cold water to the affected part, but with an *avowed*

R 2 *dread*

[* We think this opinion will meet with general opposition. Ed.]

† Fully given in a Dissertation, on the eve of being published, by the Author of this Reply.

*dread* of the remedy being worse than the disease. Hence the ground of "*Constant Reader*" triumphantly exclaiming, it has been often tried, and as often abandoned. Why has it been abandoned, if it has ever really been abandoned? Because its safety had not the warranty of my doctrine, for its general, confident, and permanent adoption.

Plagiarism is my detestation, literary robbery has a deep shade of moral turpitude; nor are the epithets "arrogant" and "*assuming*" to be deemed just or fair from a "*shrouded*" and vindictive assailant.

If to claim a right which cannot be disproved, is arrogance and assumption, then are these *gentle terms* applicable to the immortal Harvey and Jenner. Opponents were not wanting to dispute the discovery of the circulation of the blood with the former; nor have wranglers been remiss in endeavouring to garble the exclusive right of the latter to the establishment of vaccine inoculation. It was not sufficient to invalidate or even disparage the exploring merit of Harvey, that others had preceded him in imagining that the blood flowed in a circular course; nor was the public value of vaccine inoculation at all diminished in favour of Jenner's originality, by others having previously, but privately and incidentally, inoculated. Such desultory procedure was useless, and must have ever remained so, without doctrinal, systematic, and explanatory aid, to demonstrate its practical worth, and to insure its public adoption.

If, agreeably to "*Constant Reader's*" opinion of my want of originality in the refrigerant treatment of gout, the reputed efficacy of cold water, or reduced temperature, had remained on the authority of those who had recommended it, but who had not examined the nature of the disease sufficiently to perceive its perfect safety, and to entitle it to public acceptance, in what state would have been the practice at this moment? Would a gouty patient have immersed the affected limb in cold water oftener, or with more confidence, than the vaccine inoculation would have been substituted for the variolous, had the preventive efficacy of the former remained unpromulgated and unestablished by its able advocate? It may be objected the parallel does not hold. As it respects comparative originality, it does most perfectly. As it regards utility, time only can determine the question; but the probationary period which has already elapsed, since the commencement ment of my investigation of the subject, promises fully to establish the parallel also in point of utility.

If immediate relief, without the slightest inconvenience to the general health, ascribable to early cure, can entitle reduced temperature to be considered as an infallibly efficacious remedy for gout, hundreds can already attest its irresistible claim to that character; and in spite of "*shrouded*," or *unshrouded* opposition, its salutary influence will progressively gain increasing confidence, and soon prove the nature and degree of my right to originality, in scientifically authorising and establishing the topical use of reduced heat in gouty inflammation.

I now take my leave, in turn, of your "*shrouded*" correspondent, without the smallest dread of his *unshrouded* "*goose quill's*" endeavour to "*throw cold water*" on my book when it shall appear. Indeed, this wonderous witty, this "*goose quill*" threat, savours too much of *goose cackle* to be at all terrific.

Vox, et præterea nihil."

Saying no more of what is past, it may not be deemed uncharitable at parting, to desire "*Constant Reader*" to be extremely cautious in again dabbling in *mis-representation*, and what is *worse*, in being *displeased* at detection. If, after this warning, he should be again caught tripping over the sacred boundary of truth, and that in his "*propria persona*" majesty, he must expect no quarter from me; he will be deservedly consigned to literary infamy, and be for ever proscribed all claim to credit.

Until however this said "*Constant Reader*" shall think proper to quit his lurking hole, and appear in his threatened "*propria persona*," he will be too despicable a combatant for my encounter. When he shall actually skulk from his snarling retreat, he will start as fair game; and it is possible, that the *undisguised* friends of medical improvement in general, and more particularly the numerous votaries to my gouty remedy, will join me in a *hue* and *cry* against this *mighty unshrouding champion*, and laugh to scorn his impatient but malignant design of "*throwing cold water*" on a scientific and humane endeavour to establish the infallible efficacy of that fluid, in promptly and safely curing gouty inflammation.

" Spectatum admissi, risum teneatis amici."

Conscious of the rectitude of my intention, and of my indefeasible claim to that originality, which confers a right right to discovery, and which alone is worth contending for; also, fully persuaded of the important advantages already rendered to mankind by my doctrine and practice, in gouty affection, my publication on the subject will be ushered into the world without any solicitude as to its fate. Whatever may be its defects, its intrinsic merits promise long, *very long*, to survive the hostile machinations, the daring, but pitiful efforts of prejudice, calumny, malevolence, *mis-representation*, and all the other ignoble passions, whether "*shrouded*" or *unshrouded, oral* or "*goose-quilled*," "*bilious*" or "*jocular*."

"Rusticus expectat dum defluat amnis
"Labitur, et labetur in omne volubilis ævum."

I am, &c.

*Taunton, Feb.* 5, 1804. ROBERT KINGLAKE.

---

